# Bone Marrow Stromal Cells Derived MCP-1 Reverses the Inhibitory Effects of Multiple Myeloma Cells on Osteoclastogenesis by Upregulating the RANK Expression

**DOI:** 10.1371/journal.pone.0082453

**Published:** 2013-12-10

**Authors:** Zhiqiang Liu, Jingda Xu, Haiyan Li, Yuhuan Zheng, Jin He, Huan Liu, Yuping Zhong, Yong Lu, Bangxing Hong, Mingjun Zhang, Pei Lin, Juan Du, Jian Hou, Jianfei Qian, Larry W. Kwak, Qing Yi, Jing Yang

**Affiliations:** 1 Department of Lymphoma and Myeloma, Division of Cancer Medicine, Center for Cancer Immunology Research, The University of Texas M D Anderson Cancer Center, Houston, Texas, United States of America; 2 Department of Cancer Biology, Lerner Research Institute, Cleveland Clinic, Cleveland, Ohio, United States of America; 3 Department of Hematopathology, The University of Texas M D Anderson Cancer Center, Houston, Texas, United States of America; 4 Department of Hematology, The Myeloma & Lymphoma Center, Changzheng Hospital, The Second Military Medical University, Shanghai, China; University of California, San Diego, United States of America

## Abstract

Multiple myeloma (MM) cells are responsible for aberrant osteoclast (OC) activation. However, when cocultured monocytes, but not OC precursors, with MM cells, we made a novel observation that MM cells inhibited receptor activator of nuclear factor κB ligand (RANKL)-induced increase of OC differentiation, OC gene expression, signaling pathways and bone resorption activity. Our results showed that MM cells produced multiple inhibitory cytokines of osteoclastogenesis, such as IL-10, which activated STAT3 signaling and induce OC inhibition. However, cocultures of bone marrow stromal cells (BMSCs) reversed MM-induced OC inhibition. We found that MM cells increased production of MCP-1 from BMSCs and BMSC-derived MCP-1 enhanced OC formation. Mechanistic studies showed that IL-10 downregulated RANK expression in monocytes and thus, inhibited RANKL-induced OC formation. In contrast, MCP-1 upregulated RANK expression and thus, enhanced OC formation. Overall, our studies for the first time demonstrated that MM cell have inhibitory effects on osteoclastogenesis by producing inhibitory cytokines. Our results further indicate that activation of osteoclastogenesis in bone marrow requests the crosstalk of MM cells, BMSCs and their produced cytokines. Thus, our studies provide evidences that targeting bone marrow microenvironmental cells and/or cytokines may be a new approach to treating MM bone destruction.

## Introduction

Bone is a dynamic tissue that undergoes resorption and formation. Osteoclast (OC)-mediated bone resorption is crucial for normal bone homeostasis, and also plays a causative role in osteoporosis, rheumatoid arthritis, Paget disease, multiple myeloma (MM), and bone metastasis of breast cancers[1-3]. OCs, which are essentially effector cells for resorbing bone tissue, arise from hematopoietic monocytic precursors within the bone marrow cavity[4,5]. During OC differentiation, associated genes such as those for tartrate-resistant acid phosphatase (TRAP), calcitonin-related polypeptide alpha (CALCA) and CALCA receptor (CALCR), cathepsin K (CTSK), β3-integrin, and ATP-dependent proton pump subunit 18 are encoded and expressed[6,7]. Mature OCs can polarize and adhere to bone matrix, induce actin ring formation, acidify bone surface, and release osteolytic enzymes to resorb bone tissue[8]. Recent studies showed that multiple cytokines and chemokines, produced primarily by bone marrow stromal cells (BMSCs), osteoblasts, and activated immune cells, regulate osteoclastogenesis[9]. For example, receptor activator of nuclear factor kappa-B (NF-κB) ligand (RANKL) and macrophage colony-stimulating factor (M-CSF) activate OC differentiation and bone resorption activity, while RANKL decoy receptor osteoprotegerin (OPG) inhibits RANKL effects[10]. 

Bone destruction is a hallmark of MM. More than 80% of patients with MM develop osteolytic bone destruction that causes pathological fractures, bone pain, and hypercalcemia[11,12]. Recent studies showed that MM cells are responsible for activation of osteoclastogenesis. MM cells upregulate RANKL production and downregulate OPG production from BMSCs[13,14]. Moreover, MM cells express and release RANKL to the microenvironment. Increased RANKL levels and decreased OPG levels disrupt OPG/RANKL balance and induce enhanced OC differentiation and bone resorption activity[15-17]. MM cells also express multiple cytokines and chemokines, such as interleukin (IL)-3, IL-7, monocyte chemotactic protein (MCP)-1, macrophage inflammatory protein (MIP)-1α, and parathyroid hormone-related protein (PTHrP), all of which enhance OC differentiation and activity in a RANKL-dependent or -independent manner[18]. Furthermore, cocultures of MM cells have been shown to induce mature OC formation from monocyte-derived OC precursors (preOCs)[19]. However, the mechanism underlying increased OC differentiation and activity induced by MM cells remains unclear. In this study, we demonstrate for the first time that cocultures with MM cells inhibit RANKL-induced OC differentiation from monocytes but not from preOCs, and elucidate a novel mechanism whereby bone marrow osteoclastogenesis is influenced by crosstalk between MM cells, BMSCs, and derived cytokines.

## Materials and Methods

### Antibodies and reagents

Neutralization antibodies against IL-10, MCP-1, control immunoglobulin G (IgG), and recombinant human M-CSF and RANKL were purchased from R&D Systems (Minneapolis, MN). Western blot antibodies against phosphorylated or nonphosphorylated NF-κB, ERK, JNK, p38MAPK and STAT3 were purchased from Cell Signaling Technology (Danvers, MA). Antibodies against RANK were purchased from eBioscience (San Diego, CA).

### MM cell lines and primary MM cells

MM cell lines ARP-1 and ARK were kindly provided by the Arkansas Cancer Research Center, Little Rock, AR. Other cell lines were purchased from the American Type Culture Collection (Rockville, MD). Primary MM cells were isolated at the University of Texas MD Anderson Cancer Center from bone marrow aspirates of MM patients during routine clinic visits by using anti-CD138 monoclonal antibody-coated magnetic beads (Miltenyi Biotec GmbH, Auburn, CA). The study was approved by the Institutional Review Board of MD Anderson Cancer Center. All human participants provided written informed consent. All MM cells were cultured in RPMI 1640 medium (Mediatech Cellgro, VA) supplemented with 10% fetal bovine serum (FBS) and antibiotics.

### Cells cultures, cocultures, and functional assay of OCs

The method for generating OCs *in vitro* has been described previously[20]. Briefly, peripheral blood mononuclear cells (PBMCs) were obtained from healthy donor buffy coats (Gulf Coast Regional Blood Center, TX) by Ficoll-Paque (GE Healthcare, WI) density gradient centrifugation. Monocytes were isolated from the PBMCs by using anti-CD14 antibody-coated magnetic beads (Miltenyi Biotec GmbH, Auburn, CA) and cultured in α-MEM medium supplemented with 10% FBS, antibiotics, and 25 ng/mL of human M-CSF for 7 days to obtain TRAP^+^ mononuclear cells (as preOCs). PreOCs were then cultured in medium with M-CSF (25 ng/mL) and RANKL (50 ng/mL) for another 7 days to obtain multinuclear (>3 nuclei) TRAP^+^ mature OCs. The numbers of multinuclear TRAP^+^ OCs per well of 24-well plate were counted. 

In cocultures, CD14^+^ monocytes (5 × 10^5^/mL) were seeded in culture wells, and MM cells (2 × 10^5^/mL) were coseeded in culture wells or separately seeded in transwell inserts. In some cocultures, BMSCs (5 × 10^5^/mL) were also coseeded with MM cells in transwell inserts. Cells were cultured in M-CSF-containing medium with or without RANKL for 14 days. BMSCs were generated from the bone marrow aspirates of MM patients or healthy donors or were generated from human healthy fetal bones in DMEM medium supplemented with 10% FBS and antibiotics [21]. 

TRAP staining for detection of mature OCs was performed with the Leukocyte Acid Phosphatase kit (Sigma-Aldrich, MO) according to the manufacturer’s instructions. Quantitative measurement of active isoform TRAP 5b was done using BoneTRAP® Assay (Immunodiagnostic Systems Inc., Scottsdale, AZ) according to the manufacturer’s instructions.

### Quantitative real-time PCR

Total RNA was isolated with the RNeasy kit (Qiagen, MD). An aliquot of 1 μg of total RNA was subjected to reverse transcription with SuperScript II RT PCR kit (Invitrogen, CA). 1 μL of the final cDNA was applied to real-time PCR amplification with SYBR Green using the StepOnePlus real-time PCR system (Invitrogen, ABI, CA) and the following primers: 

*CTSK:* CCATATGTGGGACAGGAAGA, CCTCTTCAGGGCTTTCTCAT;

*TRAP:* AGATCCTGGGTGCAGACTTC, AAGGGAGCGGTCAGAGAATA; 

*CALCA:* TGGTGCAGAACTATGTGCAG, CTTGTTGAAGTCCTGCGTGT; 

*CALCR:* GGGAATCCAGTTTGTCGTCT, ACAAAGAAGCCCTGGAAATG; 

*IL-8:* TCCTGATTTCTGCAGCTCTG, GTCCACTCTCAATCACTCTCAG; 

*IL-10:* AGGATCAGCTGGACAACTTG, GATGTCTGGGTCTTGGTTCTC; 

*MCP-1:* CAGCCAGATGCAATCAATGCC, TGGAATCCTGAACCCACTTCT; 

*VEGF:* GAGGAGCAGTTACGGTCTGTG, TCCTTTCCTTAGCTGACACTTGT; 

*IFNγ:* GTGGTCCTCTCCTCTCTTGG, ACTGCACTGGCTATGGTGAG; 

*TPO:* CCTTCCAAGTCGGCAAATTC, ACAAAGTCCCCATTCTCCAC; 

*RANK:* GAAGAAGAAGCCAGCAGGAC, CCAGTCACATTTCCAGATGC; 

*GAPDH:* CTGGGCTACACTGAGCACC, AAGTGGTCGTTGAGGGCAATG.

### Western blotting

Cells were harvested and lysed with lysis buffer (Cell Signaling Technology, MA). Cell lysates were subjected to SDS-PAGE, transferred to a polyvinylidene difluoride membrane, and immunoblotted with antibodies against phosphorylated or nonphosphorylated NF-κB, p38, ERK, JNK, and Akt. The membrane was stripped and reprobed with anti-β-actin antibody (Sigma-Aldrich, MO) to ensure equal protein loading. Secondary antibodies conjugated to horseradish peroxidase were used for detection, followed by enhanced chemiluminescence (Pierce Biotechnology, IL) and autoradiography. 

### Flow cytometry

Cells were stained with phycoerythrin-conjugated RANK antibody (eBioscience, CA) after Fc blocking and analyzed with a FACSCalibur flow cytometer. 

### Identification of cytokines in culture medium by a cytokine array and ELISA

The supernatants (as conditioned medium) were collected from cultures of MM cells alone or BMSCs alone or cocultures of MM cells and BMSCs together in serum-free RPMI 1640 medium for 24 hours. The array (Raybio Human Cytokine Array 3 kit; RayBiotech, GA) was incubated with conditioned medium. Protein spots were detected and quantified by autoradiography according to the manufacturer’s instructions. The value of the scans was adjusted on the basis of the intensity of positive control spots on each membrane by using MultiGauge V3.0 software (Fujifilm, Japan).

In addition, cytokine levels in conditioned medium were measured by a quantitative enzyme-linked immunosorbent assay (ELISA) kit (Bethyl Laboratories, TX) according to the manufacturer’s instructions. 

### Statistical analysis

Experimental values are expressed as means ± standard errors of the means unless indicated otherwise. Statistical significance was analyzed with SPSS 10.0 software using unpaired Student’s t-tests and one-way analysis of variance. A P value ≤ 0.05 was considered statistically significant. All results were reproduced in at least three independent experiments.

## Results

### MM cells have an inhibitory effect on OC formation from monocytes

**Figure 1:**
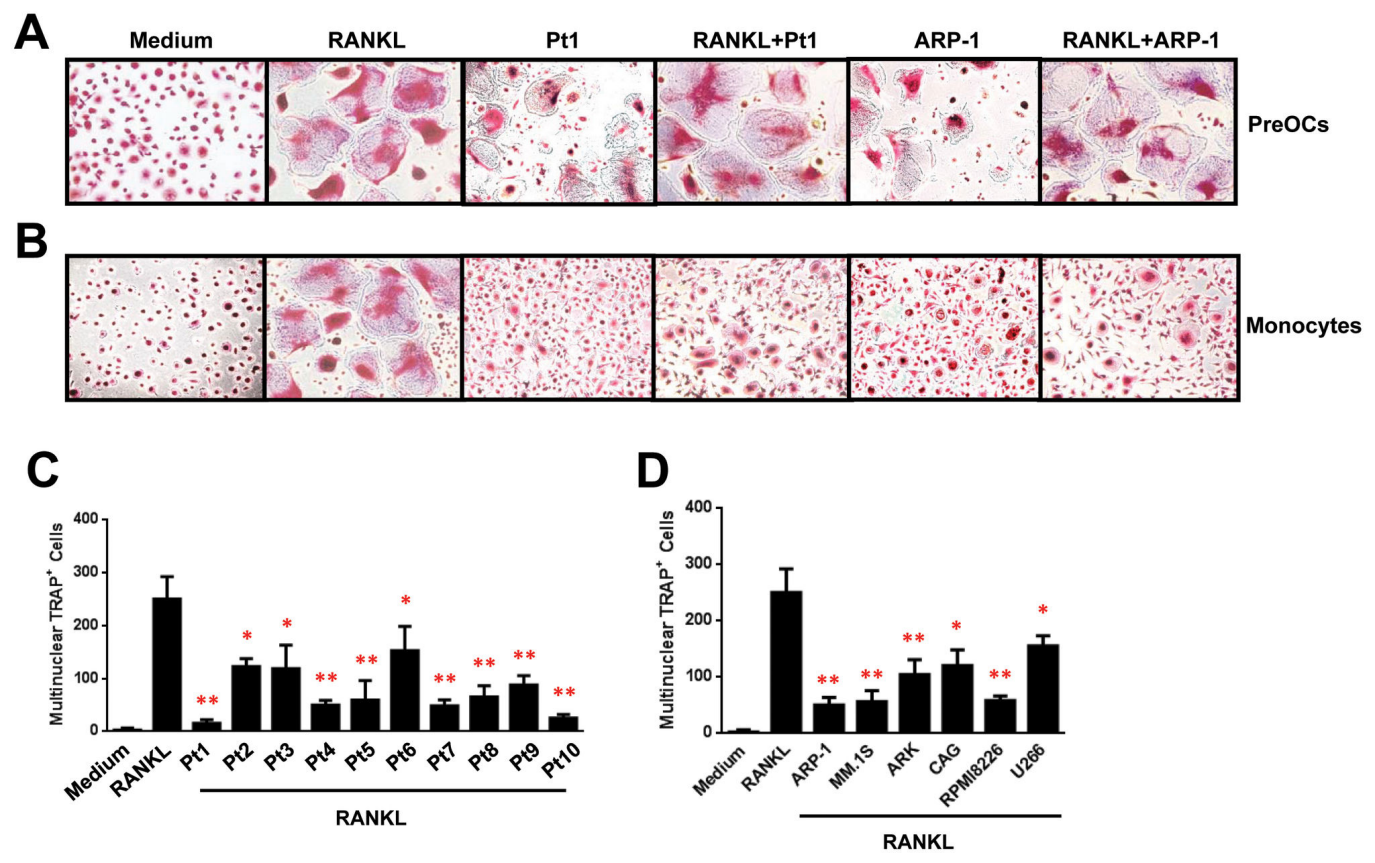
MM cells inhibit mature OC formation from monocytes. (A and B) Representative images of TRAP staining for mature OCs, defined as multinuclear TRAP+ cells, in cocultures of preOCs or monocytes without (Medium) or with primary MM cells isolated from a MM patient (Pt1) or ARP-1 cells in medium without or with RANKL (50 ng/mL). (C and D) Quantitative analysis of mature OCs generated from cocultures of monocytes with primary MM cells isolated from 10 MM patients (Pt1 to Pt10) or six MM cell lines in medium without or with RANKL. Numbers of multinuclear TRAP+ OCs per well of 24-well plate were counted. Representative results from five independent experiments are shown. *P ≤ 0.05; **P ≤ 0.01.

**Figure 2:**
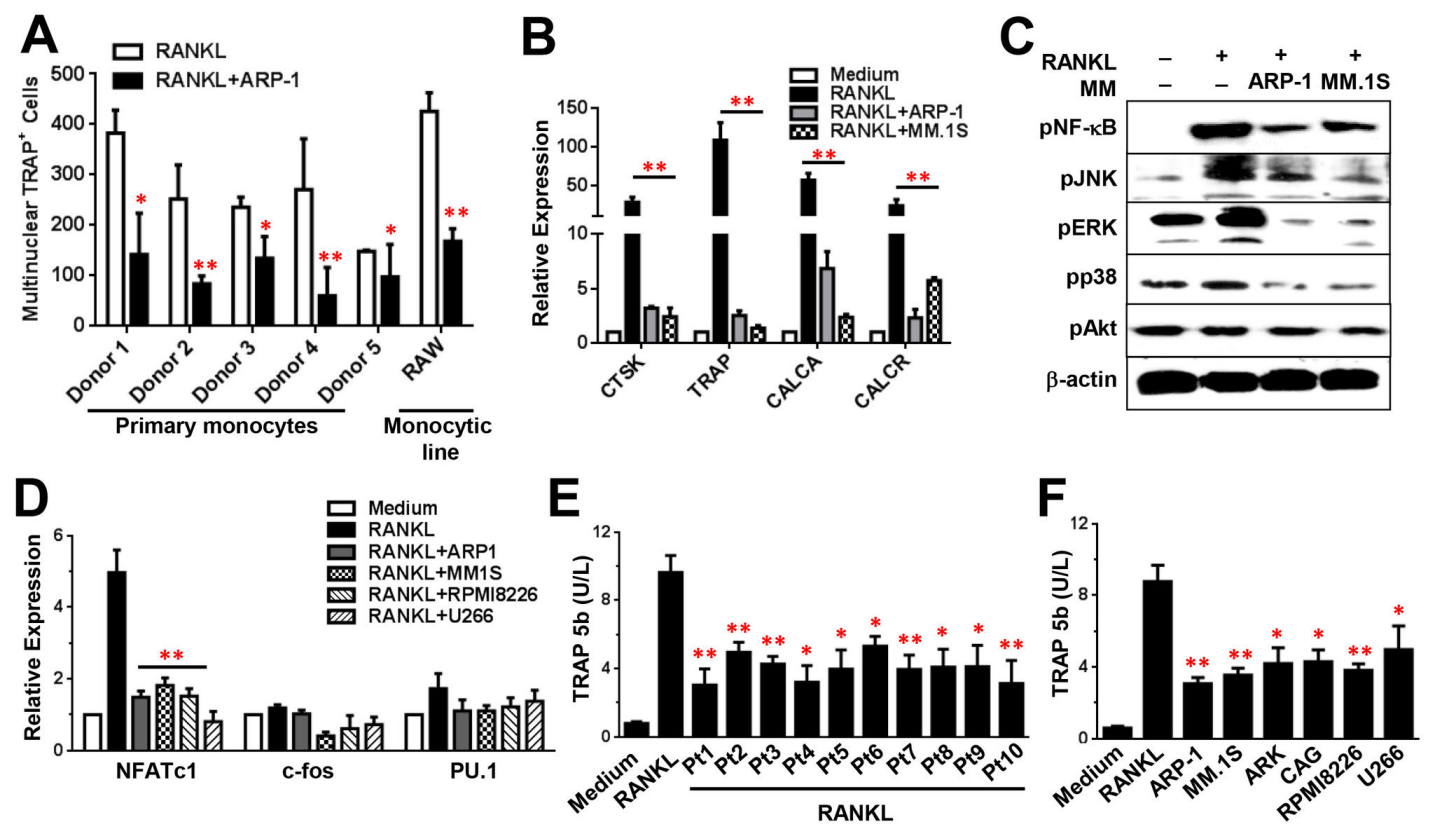
Cocultures of MM cells inhibit RANKL-induced OC differentiation and bone resorption activity. (**A**) Quantitative analysis of multinuclear TRAP^+^ OCs per well of 24-well plate generated from cocultures of monocytes, either isolated from the PBMCs of five and other (data not shown) healthy donors or generated from cocultures of myelomonocytic cell line Raw264.7 (RAW), with ARP-1 cells in medium containing RANKL (50 ng/mL). (**B**) Real-time PCR showing relative expression levels of OC differentiation-associated proteins CTSK, TRAP, CALCA, and CALCR in monocytes cocultured with ARP-1 or MM.1S cells in medium without (Medium) or with RANKL (50 ng/mL). mRNA levels of CTSK, TRAP, CALCA, and CALCR in monocytes cocultured with ARP-1 or MM.1S cells were reduced compared with those in monocytes cultured alone in medium with RANKL. (**C**) Western blot showing downregulated levels of phosphorylated (p) NF-κB, JNK, ERK, and p38, but not Akt, in monocytes cocultured with ARP-1 or MM.1S cells, compared with those in monocytes cultured alone in medium with RANKL. (**D**) Relative expression levels of NFATc1, c-fos, and PU.1 measured by real-time PCR in monocytes cocultured with ARP-1 or MM.1S cells. Cells cultured in medium without RANKL served as controls. The TRAP 5b activity of mature OCs generated from monocytes cocultured with primary MM cells from 10 MM patients (Pt1 to Pt10) (E) or MM cell lines (F) in medium with RANKL was reduced compared with that of monocytes cultured alone. Cocultured with MM cells in medium without RANKL served as control. Representative results from four independent experiments are shown. *P ≤ 0.05; **P ≤ 0.01.

Previous studies showed that RANKL, an activator of osteoclastogenesis, enhances OC differentiation and activity in vitro [22], and MM cells produce RANKL [23-25]. In lines with previous results, without RANKL, few preOCs or monocytes could differentiate into mature OCs (Figure **1A, 1B**). In the presence of RANKL, CD14^+^ monocytes, the progenitors of OCs, develop into preOCs in a 7-day culture. PreOCs further develop into mature OCs with RANKL after additional 7 days of culturing. In addition, cocultures of MM cells without RANKL induced mature OC formation from preOCs, and with presence of RANKL OC differentiation is further enhanced, in lines with previous studies[26]. Interestingly, if monocytes were cocultured with MM cells in the presence of RANKL (Figure **S1A**), the generation of mature OCs was partly inhibited (Figure **1B**). Cocultures of MM cells without RANKL could not induce OC formation from monocytes (Figure **1B**). TRAP staining showed that the addition of RANKL resulted in large numbers of mature OCs, characterized as TRAP^+^ multinuclear (>3 nuclei) cells, while cocultures of monocytes with MM cells in medium with RANKL significantly reduced the numbers of mature OCs, and without RANKL formed few of mature OCs (Figure **1B**). Cocultures of all tested primary MM cells isolated from 10 MM patients (Figure **1C**) and 6 MM cell lines (Figure **1D**) suppressed RANKL-induced OC formation from monocytes. Cocultures of MM cells and CD14^+^ monocytes, isolated from the PBMCs of either healthy donors (Figure **2A**) or MM patients (data not shown) or generated from the monocytic cell line Raw264.7 (Figure **2A**), showed similar results. We next used quantitative real-time PCR to examine the expression of OC differentiation-associated proteins CTSK, TRAP, CALCA and CALCR in monocytes, cocultured with MM cells in medium with the addition of RANKL. Addition of RANKL upregulated the mRNA levels of all tested OC-associated genes, while cocultures of ARP-1 and MM.1S cells (Figure **2B**) and other MM cells (data not shown) significantly downregulated them. 

In addition, we examined signaling pathways in monocytes during OC differentiation. We focused on NF-κB, PI3K/Akt, ERK, JNK, and p38 MAPK, because these signaling pathways are important for RANKL-induced OC differentiation[27,28]. As shown in Figure **2C**, addition of RANKL upregulated the levels of phosphorylated NF-κB, JNK, ERK and p38 MAPK but not Akt. However, cocultures of ARP-1 or MM.1S cells significantly repressed kinase phosphorylation (Figure **2C**). The levels of nonphosphorylated kinases (data not shown) and β-actin protein were unchanged. There are several hallmarks of transcription factors for OC differentiation, such as c-fos, NFATc1, and PU.1, and their relative expression levels were evaluated by real-time PCR. In our system, NFATc1 expression played a major role in RANKL-mediated OC differentiation, and its level was greatly reduced in the presence of APR-1, MM.1S, RPMI-8266, or U266 cells as shown in Figure **2D**, indicating a repressed OC differentiation by MM cells.

To assess whether the decrease in OC formation in cocultures of MM cells was accompanied by a decrease in OC bone resorption activity, we measured the levels of TRAP 5b activity, which reflects OC-induced bone resorption activity, in the same experimental settings as described above. The addition of RANKL induced high levels of TRAP 5b activity, while cocultures with MM cells, including primary MM cells from 10 MM patients (Figure **2E**) and 6 MM cell lines (Figure **2F**), significantly downregulated TRAP 5b activity. These results strongly suggest that MM cells inhibit RANKL-induced mature OC formation from monocytes. 

### MM cells produce cytokines that inhibit osteoclastogenesis

**Figure 3:**
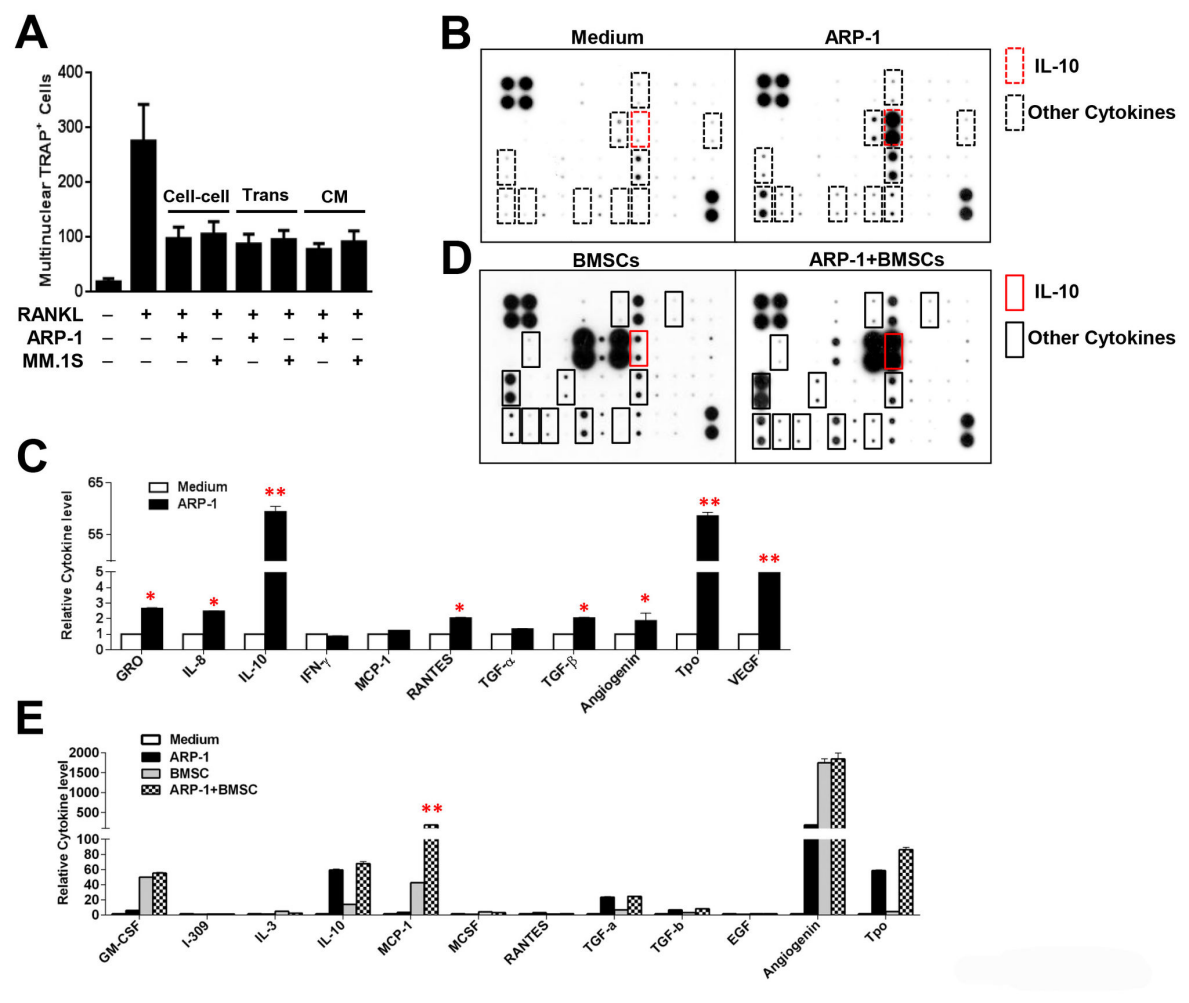
Identification of cytokines that regulate osteoclastogenesis. (**A**) Quantitative analysis of multinuclear TRAP^+^ OCs generated from monocytes cocultured with ARP-1 or MM.1S cells with cell-cell contact (cell-cell) or separated by transwell inserts (Trans) or cultures of monocytes in medium with the addition of conditioned medium (CM) and RANKL (50 ng/mL). Monocyte cultures in medium without RANKL served as controls.(**B** and **C**) Representative images (**B**) and densitometric Data (C) of a cytokine array showing the profile of cytokine expression in medium with no cell culture (Medium) and conditioned medium collected from cultures of ARP-1 cells. Increased levels of cytokines in ARP-1-conditioned medium (indicated by boxes) compared with those in medium with no cell culture are shown. (**D**) Representative images of a cytokine array showing the profile of cytokine expression in conditioned medium collected from cultures of BMSCs alone or cocultures of BMSCs and ARP-1 cells. Increased levels of cytokines in the conditioned medium from cocultures of BMSCs and ARP-1 cells (ARP-1+BMSCs) compared with those in conditioned medium from cultures of BMSCs alone are shown. In panel **B** and **D**, IL-10 was marked in red, while other cytokines were marked in black. (**E**) Densitometric data of the array showing the profile of cytokine expression in conditioned medium with no cell culture, cultures of ARP-1 cells alone, cultures of BMSCs alone, or cocultures of ARP-1 and BMSCs (MSC). Cocultured with MM cells in medium without RANKL served as control. Representative results from three independent experiments are shown.*P ≤ 0.05; **P ≤ 0.01.

To examine whether MM cells induce OC inhibition via MM cell-monocyte interaction or via MM cell-produced soluble cytokines, we used a transwell coculture system (Figure **S1B**) that disrupts MM cell-monocyte interaction but does not affect MM cell growth or the ability of MM cells to produce and release cytokines. Monocytes were seeded in culture wells and MM cells were seeded in transwell inserts in the medium with or without RANKL[29]. Transwell cocultures of monocytes with ARP-1 or MM.1S cells (Figure **3A**) or other MM cells (data not shown) retained the ability to inhibit OCs. The addition of conditioned medium, collected from cultures of ARP-1 or MM.1S cells (Figure **3A**) or other MM cells (data not shown), produced similar results. 

**Figure 4:**
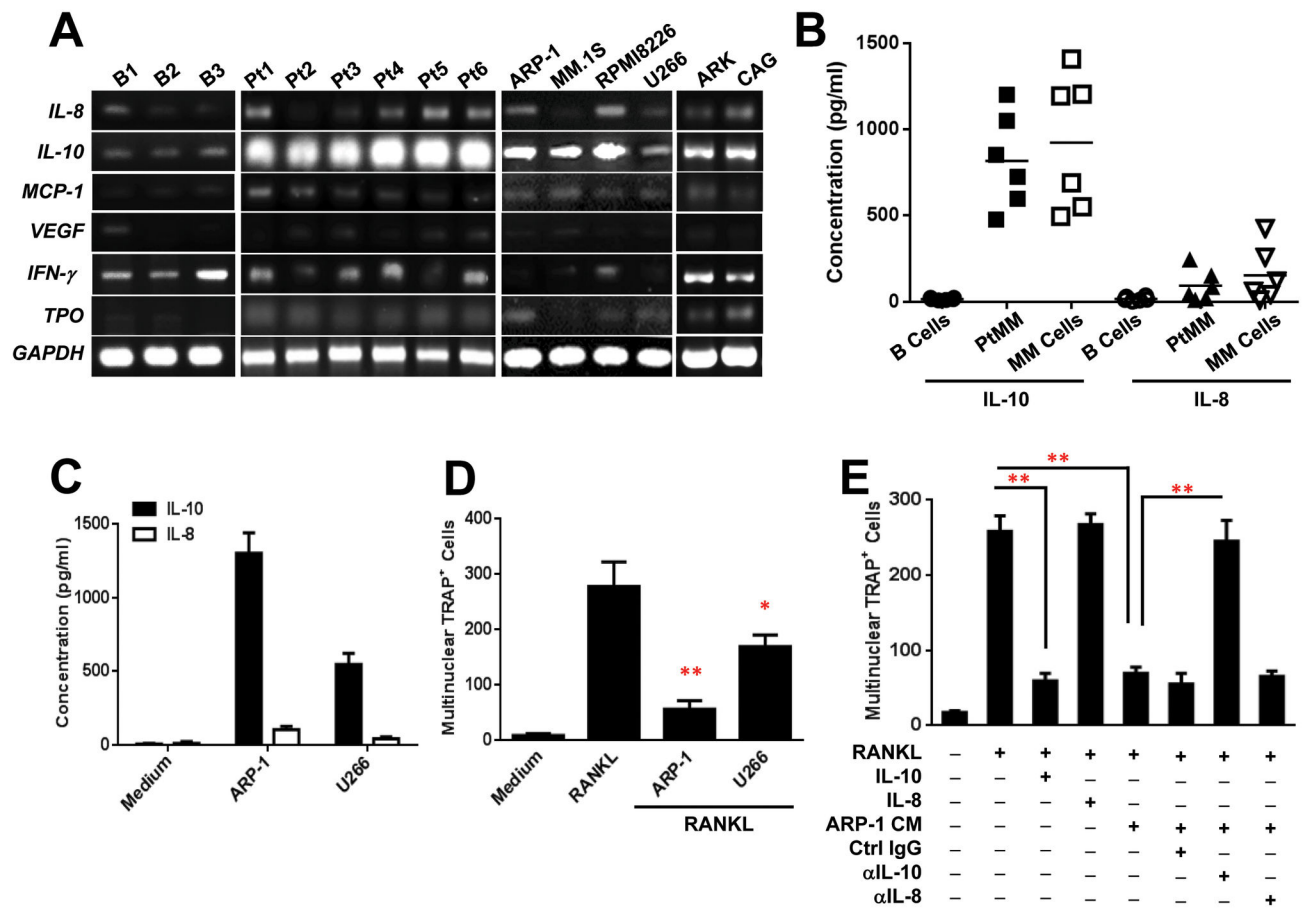
MM-derived cytokines inhibit osteoclastogenesis. (**A**) Reverse transcription PCR showing mRNA expression of IL-8, IL-10, MCP-1, VEGF, IFN-γ, and thrombopoietin (TPO) in three normal B cells isolated from healthy donors, primary MM cells isolated from six MM patients (Pt1-Pt6) or six MM cell lines (ARP-1, MM.1S, RPMI8266, U266, ARK and CAG). (**B**) Secreted IL-10 and IL-8 levels in B cells from healthy donors, primary MM cells from six MM patients (PtMM) and six MM cell lines (MM lines), determined by ELISA. (**C**) ARP-1 cells produced higher levels of secreted IL-10 than those produced by U266 cells; the levels of secreted IL-8 produced by the two cell lines were similar. (**D**) The numbers of multinuclear TRAP^+^ OCs generated from cocultures of monocytes with ARP-1 cells were lower than those generated from cocultures of monocytes with U266 cells (with the addition of RANKL for both), as determined by TRAP staining. (**E**) The numbers of multinuclear TRAP^+^ OCs generated from cultures of monocytes with the addition of RANKL and recombinant IL-10, but not IL-8, were significantly lower than those generated from cultures with the addition of RANKL only, as determined by TRAP staining. The numbers of multinuclear TRAP^+^ OCs generated from cultures of monocytes in medium containing RANKL, ARP-1-conditioned medium (CM), and neutralizing antibodies against IL-10 (αIL-10) but not IL-8 (αIL-8), were higher than those generated from the control cultures containing control IgG (Ctrl IgG). Representative results from five independent experiments are shown. *P ≤ 0.05; **P ≤ 0.01.

We next sought to determine which soluble cytokines produced by MM cells play a role in OC inhibition. Conditioned media of ARP-1 cells and media with no cell cultures were collected. An array containing specific antibodies for 48 cytokines or chemokines, the majority of which are known regulators of osteoclastogenesis, was incubated with these media. As shown in Figure 3B and 3C, the levels of eight cytokines were higher in ARP-1-conditioned medium than those in medium containing no cell cultures. Reverse transcription PCR confirmed the array results and further showed that the mRNA levels of IL-8, IL-10, MCP-1, IFN-γ, thrombopoietin and angiogenin but not VEGF, RANTES, were expressed in the majority of primary MM cells and MM cell lines (Figure **4A**). Of these cytokines, IL-10 was the most abundant in all tested MM cells (Figure **4A**). ELISA further showed that all tested primary MM cells and MM cell lines secreted high levels of IL-10. In comparison with IL-10, expression levels of IL-8 (Figure **4B**) and other cytokines (data not shown) were lower in primary MM cells from patients and MM cell lines. The addition of recombinant IL-10 (Figure **4E**), angiogenin or thrombopoietin (Figure **S2**), but not IL-8 (Figure **4E**), IFN-γ or MCP-1 (data not shown), inhibited RANKL-induced OC formation from monocytes, indicating that MM cells express and secrete several cytokines that inhibit OC differentiation. 

On the basis of these results, we focused on IL-10 and IL-8 to further verify the role and mechanism of MM-produced cytokines in MM-induced OC inhibition. We collected conditioned media of ARP-1 and U266 cells because ARP-1 cells secreted high, but U266 cells secreted low levels of IL-10, and both cell types secreted similar low levels of IL-8 (Figure **4C**). The conditioned media and RANKL were added to cultures of monocytes. Our results showed that the addition of ARP-1-conditioned medium had a greater effect on OC inhibition than the addition of U266-conditioned medium did (Figure **4D**). Furthermore, addition of recombinant IL-10, but not IL-8, to cultures of monocytes in medium containing RANKL inhibited mature OC formation (Figure **3E**). Addition of neutralizing antibodies against IL-10 to cultures of monocytes with ARP-1-conditioned medium and RANKL significantly enhanced OC formation, while addition of anti-IL-8 antibodies or control IgG had no such effect (Figure **4E**). Similar results were obtained with the addition of conditioned media of other MM cell lines and primary MM cells (data not shown). 

**Figure 5:**
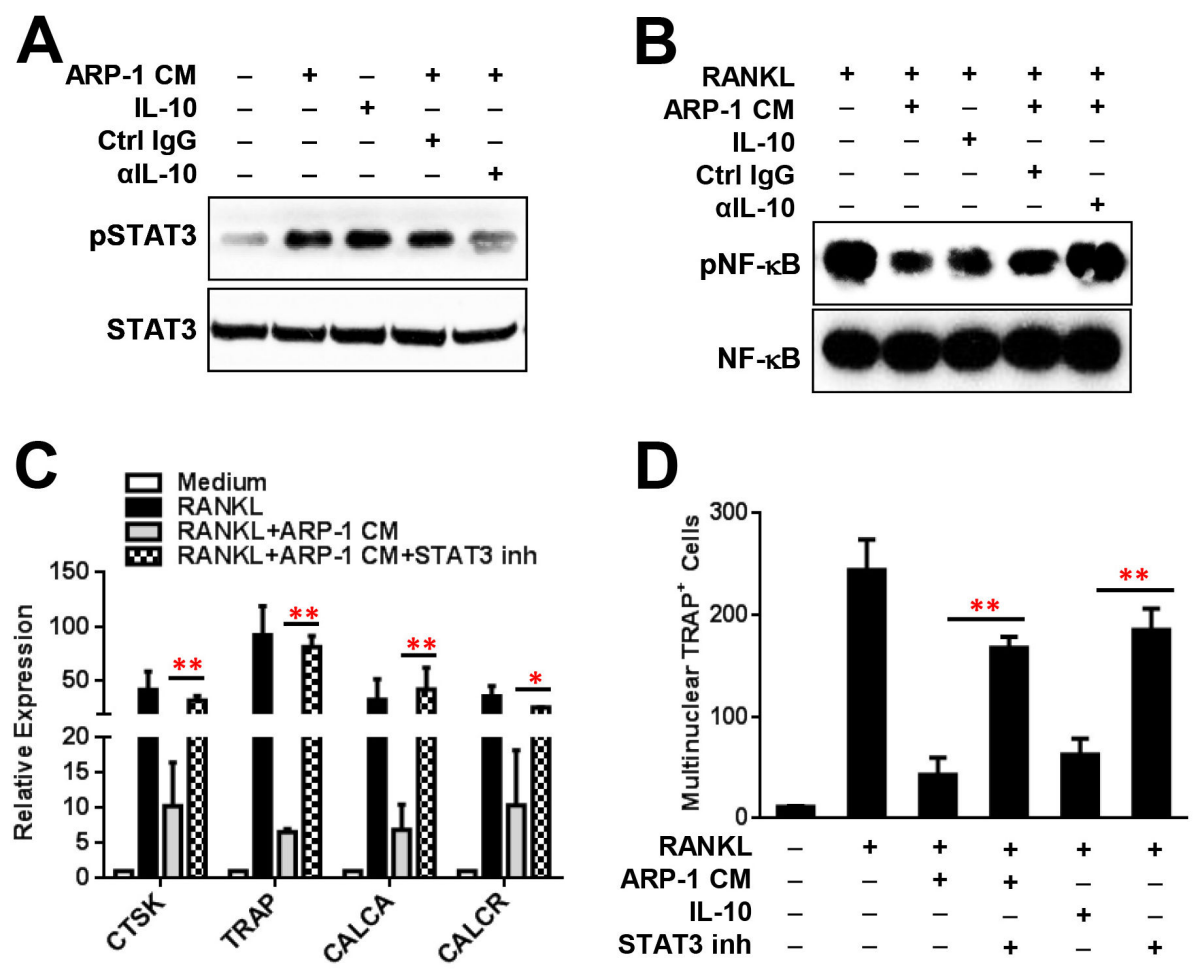
IL-10 inhibits osteoclastogenesis via STAT3 activation. (**A**) Western blot showing upregulated levels of phosphorylated STAT3 (pSTAT3) and (**B**) downregulated expression of phosphorylated NF-κB (pNF-κB) in monocytes cultured with the addition of ARP-1 conditioned medium (CM) or recombinant IL-10 and reduced levels of pSTAT3 in monocytes cultured with the addition of ARP-1 CM and neutralizing antibodies against IL-10 (αIL-10), compared with those in monocytes cultured with the addition of ARP-1 CM and control IgG (Ctrl IgG). β-actin served as a protein loading control. (**C**) Real-time PCR showing enhanced expression of OC differentiation-associated proteins CTSK, TRAP, CALCA, and CALCR in monocytes cultured with the addition of RANKL, ARP-1 CM and STAT3 inhibitors (STAT3 inh), compared with those in monocytes cultured with RANKL and ARP-1 CM only. (**D**) TRAP staining showing enhanced numbers of multinuclear TRAP^+^ OCs, generated from cultures of monocytes in medium without or with RANKL, ARP-1 CM, and/or IL-10, in the presence or absence of STAT3 inhibitors (STAT3 inh), compared with those in cultures without STAT3 inhibitors. Representative results from four independent experiments are shown. *P ≤ 0.05; **P ≤ 0.01.

Previous studies showed that STAT3 signaling pathway is activated by many inflammatory cytokines and plays a critical role in regulation of osteoclastogenesis[28]. We thus hypothesized that MM-derived cytokines such as IL-10 activates STAT3 and inhibits OC differentiation. Our results showed that addition of ARP-1-conditioned medium or recombinant IL-10 to cultures of monocytes significantly upregulated the phosphorylation of STAT3 (Figure **5A**) and downregulated phosphorylation of NF-κB (Figure **5B**). However, addition of neutralizing antibodies against IL-10, but not control IgG, to cultures of monocytes with ARP-1-conditioned medium reduced STAT3 phosphorylation (Figure **5A**). Furthermore, addition of low doses of STAT3 inhibitors significantly enhanced OC gene expression (Figure **5C**) and OC differentiation (Figure **5D**) in monocytes cultured with RANKL and conditioned medium of ARP-1 or MM.1S cells (data not shown) or recombinant IL-10. Addition of low doses of STAT3 inhibitors did not affect monocyte growth and survival (data not shown). These results clearly indicate that MM-derived cytokines such as IL-10 activate STAT3 and inhibit OC formation.

### BMSC-derived MCP-1 reverses MM-induced OC inhibition

**Figure 6:**
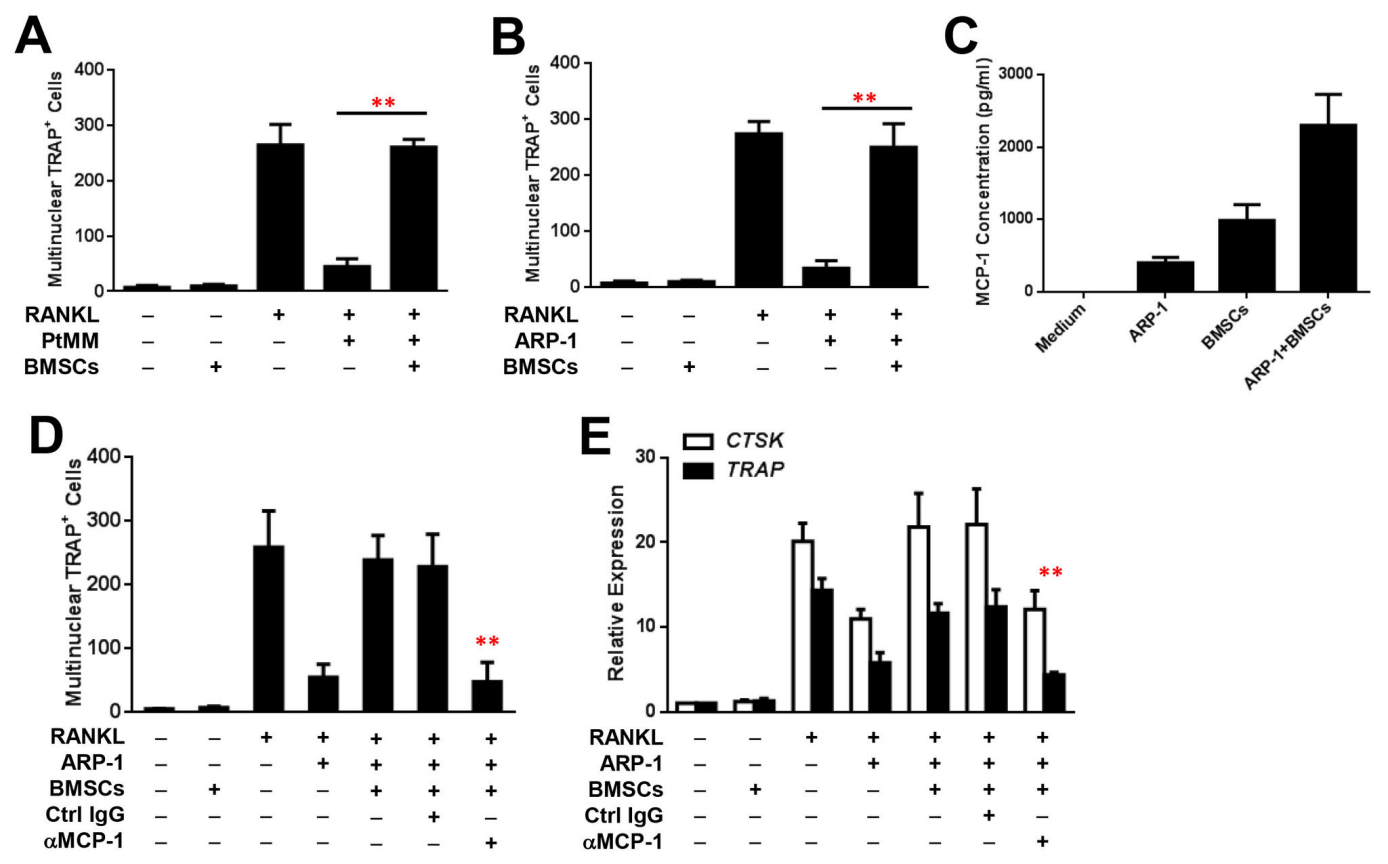
BMSC-derived MCP-1 enhances osteoclastogenesis. TRAP staining showing increased numbers of multinuclear TRAP^+^ OCs generated from cocultures of monocytes with BMSCs and either primary MM cells (PtMM) isolated from six MM patients (**A**) or ARP-1 cells (**B**) in medium with RANKL, compared with those in cultures with MM cells only or with BMSCs only. BMSCs were generated from bone marrow aspirates of MM patients or generated from human healthy fetal bones. Similar results were obtained for cocultures with MM.1S and other MM cell lines. (**C**) The levels of secreted MCP-1 in conditioned medium from cocultures of ARP-1 cells and BMSCs were higher than those from cultures of ARP-1 cells alone or cultures of BMSCs alone. Similar results were obtained for conditioned medium from cocultures of MM.1S cells and BMSCs. The numbers of multinuclear TRAP^+^ OCs per well of 24-well plate as determined by TRAP staining (**D**) and the expression levels of OC-associated proteins CTSK and TRAP, determined by real-time PCR, in OCs generated from cocultures of monocytes with ARP-1 cells and BMSCs in medium containing RANKL (**E**) were higher than those in cocultures of monocytes with ARP-1 cells only or with BMSCs only. The numbers of OCs (**D**) and the expression levels of CTSK and TRAP in OCs generated from cocultures of ARP-1 and BMSCs with the addition of RANKL and neutralizing antibodies against MCP-1 (αMCP-1) (**E**) were lower than with those from cocultures containing control IgG (Ctrl IgG). Similar results were obtained for cocultures of monocytes with BMSCs and MM.1S cells. Representative results from four independent experiments are shown. **P ≤ 0.01.

As nonmalignant BMSCs play an important role in osteoclastogenesis, we examined whether BMSCs have a role in MM-induced OC inhibition. To mimic the bone marrow microenvironment, we used a coculture system in which monocytes were seeded on culture wells, and BMSCs and MM cells, alone or together, were seeded in transwell inserts in medium with or without RANKL (Figure **S1C**). BMSCs were generated from the bone marrow aspirates of MM patients or generated from human healthy fetal bones. Our results showed that cocultures of BMSCs alone did not affect RANKL-induced OC formation (Figure **6A**). However, cocultures of monocytes with BMSCs and primary MM cells from six MM patients in medium with RANKL significantly enhanced OC differentiation (Figure **6A**) and OC gene expression (data not shown). Similar results were obtained with cocultures of BMSCs and ARP-1 cells (Figure **6B**) or MM.1S cells (data not shown). 

Furthermore, we hypothesized that soluble cytokines secreted from BMSCs might abrogate MM-induced OC inhibition. We collected conditioned media of BMSCs cultured alone, ARP-1 cells cultured alone, and BMSCs and ARP-1 cells cultured together. The media with no cell cultures served as a control. The cytokine array described above was applied. Our results showed that BMSCs secreted large amounts of cytokines into the medium (Figure **3D and 3E**). Of these cytokines, MCP-1 was produced at a high level in the conditioned medium of BMSCs cultured alone and was significantly upregulated in the conditioned medium of BMSCs and ARP-1 cells cultured together (Figure **3D and 3E**). ELISA further confirmed the array results and showed that all monocytes (Figure **S3**), BMSCs and ARP-1 cells (Figure **6C**) secreted MCP-1 with different levels, and addition of RANKL increased MCP-1 from monocytes (Figure **S3**), while in the presence of both BMSCs and ARP-1 cells, MCP-1 levels increased more than 5-fold, which was much higher than that of the cells alone (Figure **6C**). To examine the role of MCP-1 in OC activation, neutralizing antibodies against MCP-1 were added to cocultures of monocytes with MM cells and BMSCs in medium with RANKL. We observed that the addition of antibodies against MCP-1, but not control IgG, significantly reduced OC differentiation (Figure **6D**) and OC gene expression (Figure **6E**). These results indicate that the upregulation of MCP-1 production from BMSCs reverses MM-induced OC inhibition.

### Regulation of RANK expression by MM-derived IL-10 and BMSC-derived MCP-1 affects osteoclastogenesis

**Figure 7:**
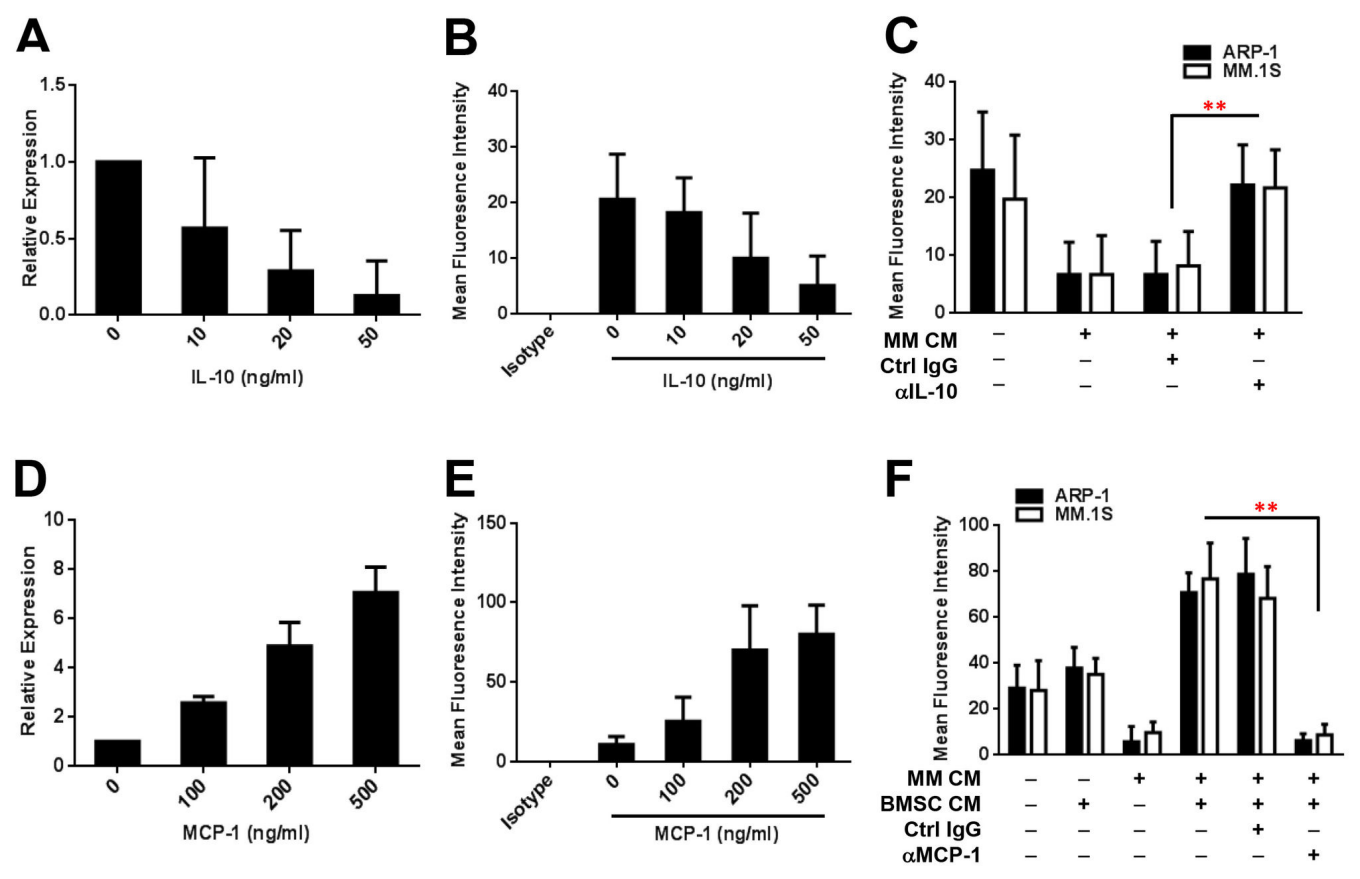
Regulation of RANK expression by IL-10 and MCP-1 affects osteoclastogenesis. Real-time PCR results for RANK mRNA levels (**A**, **D**) and flow cytometry results for RANK surface protein levels (**B**, **E**) in monocytes cultured in medium containing recombinant IL-10 (**A**, **B**) or MCP-1 (**D**, **E**). The levels of surface RANK on monocytes were reduced in cultures with the addition of conditioned medium (CM) of ARP-1 or MM.1S cells (**C**) and increased in cultures with the addition of CM from cocultures of BMSCs and either ARP-1 cells or MM.1S cells (**F**) compared with those in cultures without MM CM or in cultures without CM of BMSCs and MM cells, as determined by flow cytometry. In addition, the levels of surface RANK on monocytes were increased in cultures with the addition of CM of ARP-1 or MM.1S cells and neutralizing antibodies against IL-10 (αIL-10) (**C**) and reduced in cultures with the addition of CM of BMSC and ARP-1 or MM.1S cells and neutralizing antibodies against MCP-1 (αMCP-1) (F) compared with those in cultures containing control IgG (Ctrl IgG). Representative results from three independent experiments are shown. **P ≤ 0.01.

As RANKL binds to its receptor RANK on the progenitors of OCs and activates OC differentiation-associated signaling pathways, we wondered whether the expression of RANK in monocytes is regulated by MM-derived cytokines such as IL-10 and BMSC-derived MCP-1. Quantitative real-time PCR and flow cytometry showed that addition of recombinant IL-10 significantly downregulated mRNA (Figure **7A**) and surface protein (Figure **7B**) levels of RANK in monocytes in a dose-dependent manner. Furthermore, addition of conditioned media of ARP-1 or MM.1S cells reduced surface RANK expression, while addition of anti-IL-10 antibody, but not control IgG, to cultures of monocytes with MM-conditioned media enhanced surface RANK expression (Figure **7C**). Furthermore, addition of recombinant MCP-1 significantly enhanced mRNA (Figure **7D**) and surface protein (Figure **7E**) levels of RANK in monocytes in a dose-dependent manner. Moreover, addition of conditioned media of cocultured BMSCs and ARP-1 or MM.1S cells significantly upregulated surface RANK expression, while addition of anti-MCP-1 antibody, but not control IgG, to cultures of monocytes with conditioned media of cocultured BMSCs and MM cells reduced surface RANK expression (Figure **7F**). These results clearly indicate that regulation of RANK by IL-10 and MCP-1 affects RANKL-induced OC differentiation. 

## Discussion

This study has demonstrated for the first time that MM cells inhibit RANKL-induced OC differentiation, OC gene expression, and bone resorption activity from monocytes and repress RANKL-induced NF-κB, ERK, JNK, and p38 MAPK signaling pathways by producing several inhibitory cytokines, such as IL-10. Cocultures of BMSCs reversed MM inhibitory effects on osteoclastogenesis by upregulating MCP-1 production from BMSCs. Crosstalk between MM cells, BMSCs, and monocytes affected RANKL-induced osteoclastogenesis through regulation of RANK expression on monocytes by MCP-1 secreted by these cells and MM-derived IL-10.

Enhanced osteoclastogenesis and progressive bone-destructive lesions occur during MM cell growth in bone marrow. Previous studies showed that MM cells enhance osteoclastogenesis in vitro and in vivo. Yaccoby et al reported that MM cells upregulate mature OC formation from preOCs generated from monocytes in a 2-4 day coculture in medium with the addition of RANKL, M-CSF, and dexamethasone[26]. Other studies showed that cocultures of preOCs with MM cells induce OC formation in either the presence or the absence of BMSCs[19]. As OCs originate from monocytic lineage cells existing in the bloodstream and bone marrow, the role of MM cells in OC formation directly from monocytes was not known. This study has demonstrated for the first time that cocultures of MM cells with monocytes, but not preOCs, inhibit mature OC formation even in the presence of RANKL. However, cocultures of monocytes with both MM cells and BMSCs reversed MM inhibitory effects and restored RANKL effects on OC differentiation and activity. Osteoclastogenesis activation induced by MM cells is likely a result of interplay between monocytes, MM cells, and BMSCs within bone marrow. These findings indicate that BMSCs play an important role in osteoclastogenesis activation. 

Previous studies showed that RANKL plays a central role in OC activation. Addition of RANKL has been shown to induce OC differentiation and bone resorption activity in vitro[30,31]. BMSCs, osteoblasts and activated T cells express and release RANKL to the bone marrow microenvironment [32,33]. Moreover, MM cells stimulate BMSC production and release of RANKL and other cytokines, such as MIP-1α, VEGF, TNFα, MCP-1, stromal cell-derived factor-1, IL-3, and IL-7, into the bone marrow microenvironment, and in turn these cytokines enhance OC differentiation and activity in a RANKL-dependent or -independent manner[34-36]. BTK inhibitor PCI-32765 has been shown to be able to block RANKL/M-CSF-induced phosphorylation of Btk and downstream PLC-γ2 in OCs, and diminish TRAP 5b and bone resorption activity [37]. In addition, MM cells also produced multiple cytokines that involve in OC activation, such as PTHrP. Elevated levels of PTHrP were reported in a number of clinical myeloma cases, and recent studies by Cafforio et al. have found that both the full-length molecule and NH_2_-terminal fragment of PTHrP reinforces the production of osteoclastogenic factors RANKL and MCP-1, thus activate osteoclastogenesis [38]. On the other hand, several cytokines play a negative role in osteoclastogenesis. For example, OPG has been shown to disrupt RANKL-induced OC activation[39]. In patients, production of OPG from BMSCs is reduced by MM cells[40]. Our studies identified several OC-inhibitory cytokines expressed and secreted by MM cells, such as IL-10, angiogenin, and thrombopoietin, which inhibited osteoclastogenesis. Antibodies against these cytokines enhanced OC formation. These findings indicate that MM cells express and secrete both stimulatory cytokines such as RANKL and inhibitory cytokines such as IL-10, angiogenin, and thrombopoietin, which work in a complementary manner in regulating osteoclastogenesis in bone marrow.

IL-10 is an inflammatory cytokine involved in immunosuppression[41]. Previous studies showed that IL-10 stimulates RANKL production from BMSCs in vitro and activates osteoclastogenesis in vivo[42]. However, IL-10 upregulates STAT3 and downregulates NF-κB, leading to OC inhibition[43]. Our results showed that all tested MM cell lines and primary MM cells from MM patients produced high levels of IL-10. Addition of anti-IL-10 antibody enhanced OC formation in cocultures of monocytes with MM cells via STAT3 activation. Furthermore, our study elucidates a novel mechanism that MM-derived IL-10 inhibited RANKL-induced OC differentiation from monocytes by downregulating RANK expression. These results indicate that there is a feedback loop in IL-10-induced osteoclastogenesis, in which IL-10 upregulates RANKL production, but downregulates RANK expression and inhibits RANKL-induced OC differentiation and activity. 

MCP-1 is a chemokine that recruits monocytes, T cells, and dendritic cells to sites of infection or tissue injury. Previous studies have shown that MCP-1 promotes the fusion of hematopoietic precursors to mature OCs [44], rescues granulocyte macrophage colony-stimulating factor-induced OC inhibition [44], and determines OC activity by binding to receptor CCR2 [45]. MCP-1 can be secreted from monocytes [44] and BMSCs [46], and its levels are upregulated by RANKL during osteoclastogenesis [44,47]. Moreover, we previously found that MM cells also secreted low levels of MCP-1, and MM-derived MCP-1 involved in OC differentiation [20]. ELISA results showed that MM cells ARP-1 produced around 500 pg/ml of MCP-1in cultured media. However, we also observed that in the presence of both BMSCs and ARP-1 cells, the levels of MCP-1 in cultured media increased more than 5-fold, which is much higher than that of ARP-1 cells alone or BMSC alone, indicating a synergistic effect from the combination presence of BMSCs and MM cells.

The OC inhibition induced by MM cells, MM-derived IL-10, or other MM-derived inhibitory cytokines was quenched in the presence of BMSCs. In vitro cocultures of monocytes with MM cells and BMSCs created a mimicking of MM-bearing bone marrow in vivo. In cocultures of these cells, the osteoclastogenesis-suppressive function of IL-10 was reversed by BMSC-derived MCP-1. Mechanistic study showed that addition of IL-10 downregulated RANK expression in monocytes, while addition of anti-IL-10 antibody to cocultures of monocytes and MM cells enhanced RANK expression. In contrast, addition of MCP-1 upregulated RANK expression, while addition of anti-MCP-1 antibody to cocultures of monocytes, MM cells, and BMSCs reduced RANK expression. These results indicate that the regulation of RANK levels in monocytes by MM-derived IL-10 and BMSC-derived MCP-1 affects activation of osteoclastogenesis by RANKL. However, the integrative mechanism through which the IL-10- and MCP-1-activated signaling pathways regulate RANK gene and surface protein expression warrant further investigation. 

## Supporting Information

Figure S1 **Coculture systems.** (**A**) Monocytes (5 × 10^5^/mL) and MM cells (1 × 10^5^/mL) coseeded in culture wells; (**B**) monocytes (1 × 10^5^/mL) seeded in culture wells and MM cells (5 × 10^5^/mL) seeded in transwell inserts; (**C**) monocytes (5 × 10^5^/mL) seeded in culture wells and MM cells (1 × 10^5^/mL) and BMSCs (5 × 10^5^/mL) coseeded in transwell inserts. Cells were cultured in minimum essential medium alpha supplemented with 10% FBS, antibiotics, human M-CSF (50 ng/mL), and RANKL (50 ng/mL) for 14 days.
(DOCX)

Figure S2 **Effect of angiogenin or thrombopoietin on RANKL-induced OC differentiation.** In the presence of 100 ng/ml angiogenin or 20 ng/ ml thrombopoietin, OC differentiation was repressed, as measured by the numbers of multinuclear TRAP^+^ cells per well/24-well plate (**A**) and levels of TRAP 5b by ELISA (**B**).
(DOCX)

Figure S3 **Monocytes secret MCP-1 and RANKL stimulates monocyte secretion of MCP-1.** The levels of secreted MCP-1 were examined with ELISA. Cultured CD14^+^ monocytes were isolated from the PBMCs by using anti-CD14 antibody-coated magnetic beads and were incubated with or without (Medium) 50ng/ml of RANKL for 24 hours. ELISA showed that monocytes secret MCP-1 and the MCP-1 level was higher in medium of cultured monocytes with RANKL than those of monocytes without RANKL.
(DOCX)
