# Supplementary material for: Bone Marrow Stromal Cells Derived MCP-1 Reverses the Inhibitory Effects of Multiple Myeloma Cells on Osteoclastogenesis by Upregulating the RANK Expression
Source: PLoS One. 2013 Dec 10;8(12):e82453. doi: 10.1371/journal.pone.0082453 (PMC3858321; doi:10.1371/journal.pone.0082453)

**Figure S1. Coculture systems.** (**A**) Monocytes (5 × 10^5^/mL) and MM cells (1 × 10^5^/mL) coseeded in culture wells; (**B**) monocytes (1 × 10^5^/mL) seeded in culture wells and MM cells (5 × 10^5^/mL) seeded in transwell inserts; (**C**) monocytes (5 × 10^5^/mL) seeded in culture wells and MM cells (1 × 10^5^/mL) and BMSCs (5 × 10^5^/mL) coseeded in transwell inserts. Cells were cultured in minimum essential medium alpha supplemented with 10% FBS, antibiotics, human M-CSF (50 ng/mL), and RANKL (50 ng/mL) for 14 days.


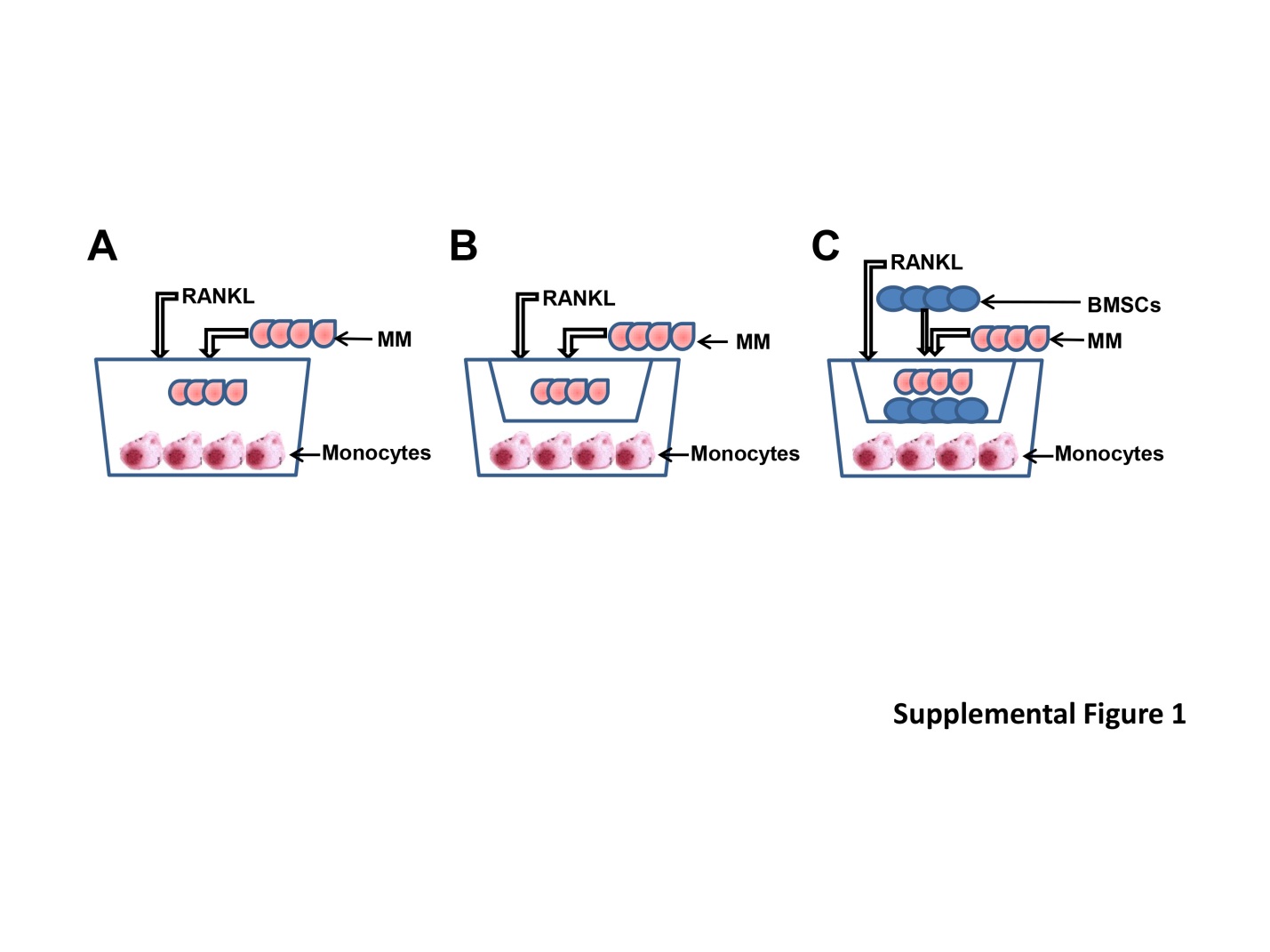

Supplement: Figure S1 — Coculture systems. (A) Monocytes (5 × 10^5^/mL) and MM cells (1 × 10^5^/mL) coseeded in culture wells; (B) monocytes (1 × 10^5^/mL) seeded in culture wells and MM cells (5 × 10^5^/mL) seeded in transwell inserts; (C) monocytes (5 × 10^5^/mL) seeded in culture wells and MM cells (1 × 10^5^/mL) and BMSCs (5 × 10^5^/mL) coseeded in transwell inserts. Cells were cultured in minimum essential medium alpha supplemented with 10% FBS, antibiotics, human M-CSF (50 ng/mL), and RANKL (50 ng/mL) for 14 days. (DOCX) [file pone.0082453.s001.docx]
