# Supplementary material for: Bone Marrow Stromal Cells Derived MCP-1 Reverses the Inhibitory Effects of Multiple Myeloma Cells on Osteoclastogenesis by Upregulating the RANK Expression
Source: PLoS One. 2013 Dec 10;8(12):e82453. doi: 10.1371/journal.pone.0082453 (PMC3858321; doi:10.1371/journal.pone.0082453)

**Figure S2**. **Effect of angiogenin or thrombopoietin on RANKL-induced OC differentiation**. In the presence of 100 ng/ml angiogenin or 20 ng/ ml thrombopoietin, OC differentiation was repressed, as measured by the numbers of multinuclear TRAP^+^ cells per well/24-well plate (**A**) and levels of TRAP 5b by ELISA (**B**).


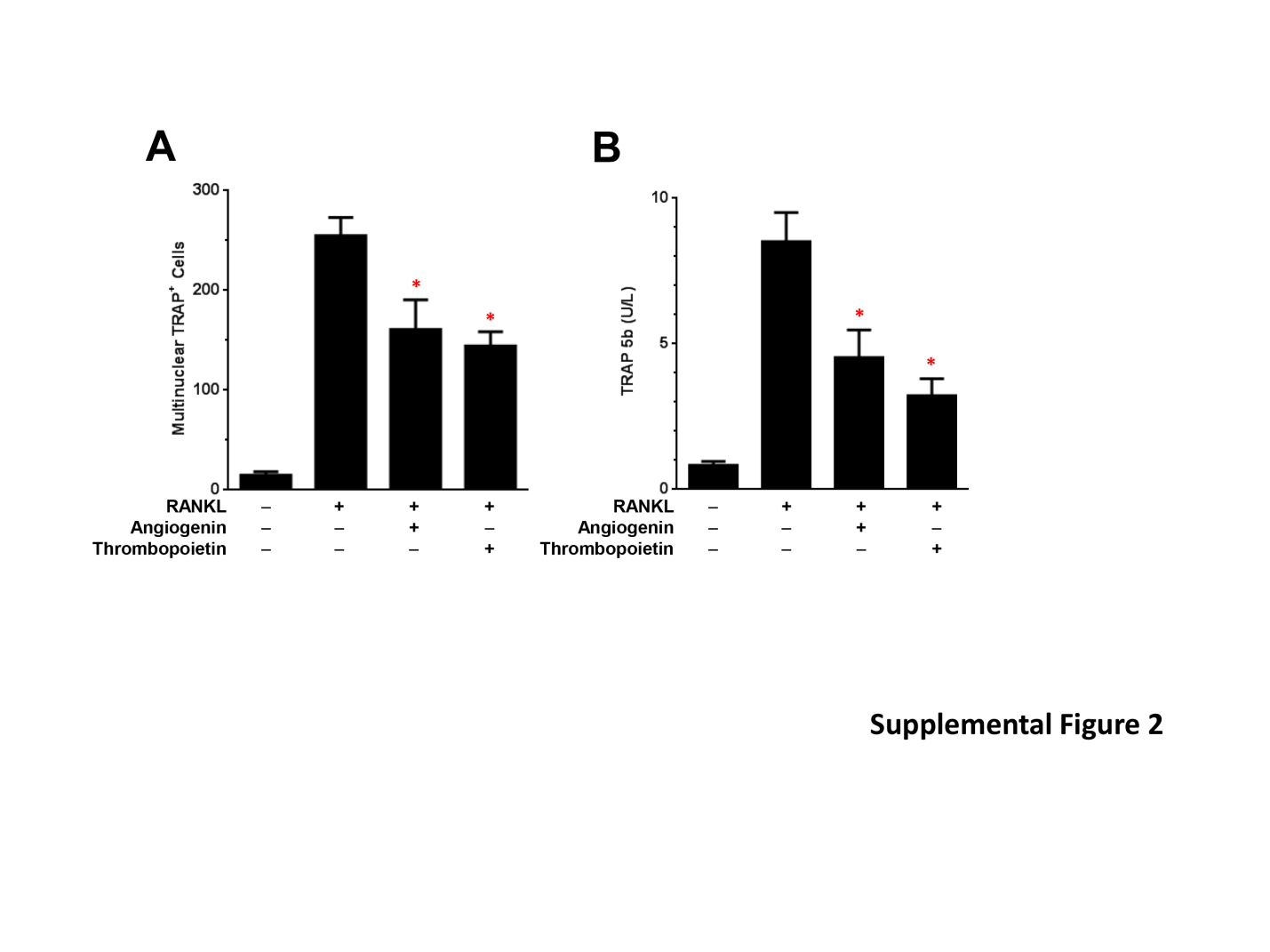

Supplement: Figure S2 — Effect of angiogenin or thrombopoietin on RANKL-induced OC differentiation. In the presence of 100 ng/ml angiogenin or 20 ng/ ml thrombopoietin, OC differentiation was repressed, as measured by the numbers of multinuclear TRAP+ cells per well/24-well plate (A) and levels of TRAP 5b by ELISA (B). (DOCX) [file pone.0082453.s002.docx]
