# Supplementary material for: Bone Marrow Stromal Cells Derived MCP-1 Reverses the Inhibitory Effects of Multiple Myeloma Cells on Osteoclastogenesis by Upregulating the RANK Expression
Source: PLoS One. 2013 Dec 10;8(12):e82453. doi: 10.1371/journal.pone.0082453 (PMC3858321; doi:10.1371/journal.pone.0082453)
Supplement: Figure S3 — Monocytes secret MCP-1 and RANKL stimulates monocyte secretion of MCP-1. The levels of secreted MCP-1 were examined with ELISA. Cultured CD14+ monocytes were isolated from the PBMCs by using anti-CD14 antibody-coated magnetic beads and were incubated with or without (Medium) 50ng/ml of RANKL for 24 hours. ELISA showed that monocytes secret MCP-1 and the MCP-1 level was higher in medium of cultured monocytes with RANKL than those of monocytes without RANKL. (DOCX) [file pone.0082453.s003.docx]

**Figure S3. Monocytes secret MCP-1 and RANKL stimulates monocyte secretion of MCP-1.** The levels of secreted MCP-1 were examined with ELISA. Cultured CD14^+^ monocytes were isolated from the PBMCs by using anti-CD14 antibody-coated magnetic beads and were incubated with or without (Medium) 50ng/ml of RANKL for 24 hours. ELISA showed that monocytes secret MCP-1 and the MCP-1 level was higher in medium of cultured monocytes with RANKL than those of monocytes without RANKL.

**
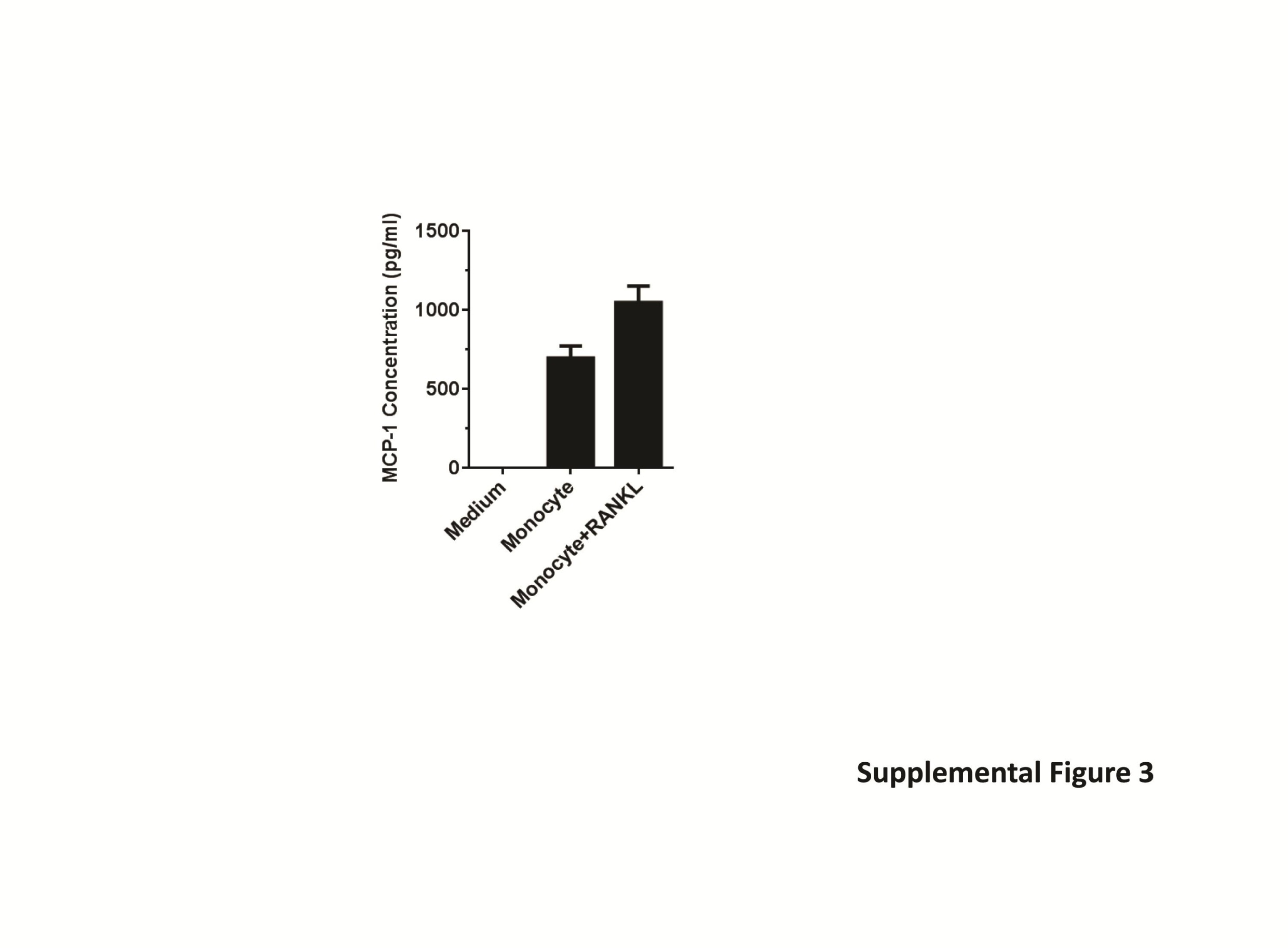
**
